# Impact of tobacco industry and other corporations in the defeat of the 1994 Clinton health care plan

**DOI:** 10.1186/s12889-017-4501-7

**Published:** 2017-06-21

**Authors:** Michael Givel

**Affiliations:** Department of Political Science, The University of Oklahoma, 455 West Lindsey, Room 215, Norman, OK 73019 USA

**Keywords:** Bill Clinton, 1994 health care reform, Corporate lobbying, Tobacco industry, Congress, White House

## Abstract

**Background:**

The primary reason cited by many scholars for the defeat of the Clinton Administration’s 1994 health care reform bill has long been identified as Health Insurance Association of America and National Federation of Independent Businesses opposition to the bill. Given this predominant consensus combined with sizeable proposed funding for the bill by a large tobacco product tax, this manuscript examined what the tobacco industry’s role was in whole or part in defeating the Clinton health care bill.

**Methods:**

This research occurred through crosschecking internal tobacco industry documents and Clinton White House documents.

**Results:**

Prior to the passage of the bill, the tobacco industry accepted a compromise of 45 cents per pack increase phased in over five years. Due to this compromise, the industry or third party allies had no role in the ultimate defeat in the bill.

**Conclusions:**

The primary reason for the bill’s ultimate defeat was general business (but not tobacco industry and third party ally) opposition, the bill running out of time, and conflicting bills. Secondary reasons for the bill’s defeat included issues with: employer mandates, high taxes on insurance plans, impacts on medical research and education, Congressional attention to other issues, election year politics, and possible future excise tax possibilities.

## Background

What role did the tobacco industry and other corporations play in contributing to the defeat of the Clinton administration’s effort to reform the U.S. health care system under the 1994 Health Security Act (HSA)? The impetus for health care reform was the containment of soaring health care costs for employees during the 1980s that was being paid and absorbed by numerous employers [1–4]. The major provisions of the proposed HSA included universal coverage except undocumented persons, state and regional private health insurance purchasing alliances, subsidies to individuals to pay for health care, minimum health care benefit packages approved by federal law, and regulatory oversight of premium price increases by private insurers [1–5].

The primary reason the bill was defeated according to many scholars was vigorous business opposition, in particular, from the Health Insurance Association of America (HIAA) and National Federation of Independent Business (NFIB) [1, 3, 6]. According to Quadango regarding medium health insurance company opposition:

‘The purchasing alliances would be similar to the corporate purchasing coalitions of the 1980s and dominated by the five largest health insurers: Aetna, Prudential, MetLife, Cigna, and Travelers. However, smaller specialty firms stood to lose 30 to 60 percent of their business, and insurance agents would be put out of business’ [2].

The HIAA also had a major ally in small businesses represented by NFIB who opposed employer mandates and tax increases [2]. Numerous scholars have concluded that a range of ancillary factors also led to the 1994 health care bill’s defeat. These factors included conservative opposition to higher taxes and more government regulation, dragging out the process allowing the opposition to organize, election year politics, and health industry opposition to the plan [4, 7–10]. Another key opponent of the bill was the Pharmaceutical Research and Manufacturer’s Association, which argued rising drug costs due to the legislation would stifle research, prevent new drugs from entering the market, and were already reasonably priced [11].

Almost all scholarly research has concluded that the tobacco industry has had little or no influence in defeating the 1994 health care bill [7–10]. However, so far, one peer reviewed article has concluded that the tobacco industry did have an important impact [12]. The overall conclusion of the role and impact of the tobacco industry’s effort to defeat the health care according to the authors of this paper was, ‘…the tobacco industry credited itself with a significant role for the defeat of the bill [12]. This was accomplished, according to this article, by mobilizing ideologically diverse third parties to oppose the Clinton plan. As Tesler and Malone [12] wrote:

The tobacco industry’s success in mobilizing opposition to the Clinton plan among ideologically diverse constituencies underscores the challenge of overcoming corporate efforts to obstruct health care reform [12]

The authors also noted, in particular, that the tobacco industry opposed a higher tobacco excise tax funding provision to pay for the 1994 health care legislation and noted that multiple other factors contributed to the bill’s defeat [12]. Given this difference between most scholars who argue it was primarily the HIAA and NFIB and the one paper concluding the tobacco industry has a significant role and impact in defeating the Clinton health reform bill, this paper will further analyze and clarify this question.

In order to shed new light on this question this paper will utilize tobacco industry internal documents as well as Clinton White House documents to determine what role the tobacco industry had in defeating the 1994 health care legislation. This paper is the first to use Clinton White House records to crosscheck tobacco industry documents to determine the role, if any, of the tobacco industry in the defeat of Clinton’s 1994 Health Security Act.

## Methods

This paper examined all of the fully quoted text in the tobacco documents between November 1993 and November 1994 that directly describes significant final conclusions on why the health care bill was defeated. Significant final conclusion for the paper is defined as, the final summarization as contained in a document of why the health care bill was defeated. This time period was chosen because the bill was introduced in Congress on 22 November 1993 and encompassed the months in which Congress deliberated on the bill, which was defeated in September 1994 as well as any analyses after the bill was defeated [1, 3, 7–10].

Data collection was performed by searching the more than 80 million pages of previously internal tobacco industry documents obtained in the settlement of *State of Minnesota,* et al. *v. Phillip Morris,* et al. (No.C1–94-8565, 2nd District, Minnesota) and other subsequent litigation against the tobacco industry. Under the terms of the settlement, five tobacco companies, a tobacco trade association, and a tobacco industry research council established searchable web sites for these documents. This material was accessed on the Internet at the University of California, San Francisco Legacy Tobacco Documents web site, which has integrated the various tobacco industry web sites (https://www.industrydocumentslibrary.ucsf.edu/tobacco/). Using the search term “health care” and Clinton within the specified time period there were 4091 “hits.” This Boolean search phrase was selected so the search engine would identify all available documents from the entire text of the tobacco documents related to the 1994 Clinton health bill. Ultimately, 26 files provided pertinent references that directly described significant tobacco company final conclusions on why the bill was defeated.

A search of the tobacco documents was also conducted to determine if the tobacco industry had quietly worked through third party allies to defeat the bill (Table 1). Often, corporate lobbying can be oblique with indirect lobbying by an industry like tobacco being conducted indirectly through other allied groups that have been instrumental in ultimately defeating or enacting a bill. Based on prior research, a corporate lobby that opposed the bill entirely was the HIAA. This paper also reveals from the Clinton era documents that a coalition of corporate lobbies opposing the bill included: Pharmaceutical Research and Manufacturer’s Association, U.S. Chamber of Commerce, NFIB, National Association of Manufacturers, Business Roundtable, Association of Private Pension and Welfare Plans, and the Employee Retirement Income Security Act Industry Committee [13]. The following table summarizes the search results.

**Table 1:** Legacy tobacco document search results for relevant tobacco industry and corporate ally documents from 22 November 1993 to 31 December 1994 to defeat Clinton health care bill

Search terms	Number of hits	Relevant documents
“Health Insurance Association of America” and Clinton	38	0
“National Federation of Independent Businesses” and Clinton	49	0
“U.S. Chamber of Commerce and Clinton;	0	0
“National Association of Manufacturers” and Clinton	65	0
“Association of Private Pension and Welfare Plans” and Clinton	3	0
“Pharmaceutical Research and Manufacturer’s Association” and Clinton	0	0
“*Employee Retirement Income Security Act Industry Committee”* and Clinton	0	2

Data collection was performed for Clinton Presidential documents by searching online declassified documents that included references to tobacco and the Health Security Act of 1994 that directly described significant final conclusions on why the health care bill was defeated. This material was accessed on the Internet at the Clinton Digital Library web site at: http://clinton.presidentiallibraries.us/collection-tree. The approach used in the examination of these documents provided by an archivist to this author at the Clinton Library included examining: the “Domestic Policy Council Collections - http://clinton.presidentiallibraries.us/collections/show/24 - especially Chris Jennings HSA collection (http://clinton.presidentiallibraries.us/collections/show/51) Elena Kagan Collection - http://clinton.presidentiallibraries.us/collections/show/34. Previously Restricted Documents Collections http://clinton.presidentiallibraries.us/collections/show/43. The search term suggested by the archivist and used was: “tobacco and Health Security Act” producing 49 “hits.” This Boolean search phrase was selected so the search engine would identify all available documents related to final conclusions why the 1994 Clinton bill was defeated.

Empirical coding of the universal set of qualitative data in the tobacco and Clinton documents that referenced significant final conclusions for the defeat of Clinton health bill and possible tobacco industry linkages with corporate third parties was accomplished using NVIVO 9.0 qualitative data analysis software produced by QSR International Inc. All pertinent documents that were uncovered are cited in this paper and included in the reference section. After a content analysis of the tobacco documents and the Clinton White House occurred to ascertain the role, if any, of the tobacco industry and third party organizations it utilized in the defeat of the Clinton health care bill, the major conclusion from these two sets of documents was crosschecked with each other. The method used in this archival research paper to counter subjectivity bias is the audit trail approach. As noted above this provides a clear depiction of the research steps taken including the reporting of findings, which a third party can replicate and analyze for themselves [14].

## Results

Based on the coded internal tobacco industry documents between January 1993 and November 1994 that directly reference the tobacco tax increase to fund the health care bill, there were a myriad of complex reasons from the perspective of tobacco firms that led to the defeat of the 1994 health care legislation (Fig. 1) [15–41]. With respect to the role of the tobacco industry in the health care bill’s defeat, from January to June 1994, several references in the tobacco documents indicate that the industry originally opposed *any* tobacco tax increase [24–26, 38, 39]. This was due to an initial proposal by the Clinton Administration of taxing tobacco at two dollars a pack, which was also reflected in a January 1993 report: *Tobacco Use: An American Crisis* emanating from a conference co-sponsored by the American Medical Association, City of Hope National Medical Center, CDC, Coalition on Smoking or Health, Memorial Sloan-Kettering Cancer Center, and University of Texas Anderson Cancer Center [42]. A May 1994 Philip Morris document also indicated that prior to July 1994 Philip Morris was involved in a corporate coalition with ‘National Association of Manufacturers, Citizens for Tax Justice, and others’ to oppose any tobacco tax increase [43].

However, eight references in tobacco documents from July to September 1994 indicate that the industry accepted a political compromise based on 45 cents per pack tobacco increase phased in over five years [19–22]. As a representative Philip Morris internal document triumphantly concluded:

‘…it is no coincidence that the politics have forced the level down from the proposed one time increase of $2.00 per pack to the current proposal of a 45¢ increase phased in over five years. While our opponents never miss a chance to promote their positions with the media, we have aggressively but quietly worked the pressure points because, in the end, it is votes that count not ink. In many ways, the excise tax fight has been a case study of combining our Washington talent and experience with our corporate resources to achieve the best possible outcome for the company’ [21].

The industry’s strategy to scale back the initial $2.00 a pack proposal to a .45-cent increase over five years included the following:

‘Our federal excise tax strategy was based on several key assumptions. (C) First, a far less ambitious and consequently less costly package would give the Tax Committees more flexibility in terms of financing. This would give us room to maneuver on the tobacco tax. To impact that debate, we organized intense grassroots efforts targeting swing Democratic Members arguing against wholesale changes to the health care system. Efforts at the grassroots level eventually deadlocked key Committees and forced the Congress to retreat from the costly Clinton plan.

(C) And, with the House and Senate leadership initially depending on Democrats alone to pass legislation, the Democratic tobacco-voting bloc's leverage was significantly increased. We constantly worked to maintain the solidarity and intensity of their efforts. And as southern Democrats, their reelection difficulties were not lost on their leadership.

(C) Finally, our strategy was to win the issue in the House by concentrating our activities in the House Ways and Means Committee which would act first on the tax. Winning in the House would provide Senate Majority Whip Wendell Ford with an 'anchor' and a greater ability to negotiate in the Senate where our support is much weaker’ [21].

This was accomplished in the following manner^1^:

‘Throughout the year, our lobbying activities have included organizing Hill activities for labor and tobacco grower organizations, creating a grower advertising campaign, working with anti-tax groups to oppose excise taxes, funding several economic analyses suggesting alternative financing mechanisms, and developing and implementing an anti-excise tax grassroots campaign lead by those interests who have a direct economic stake in the outcome’ [21].

Since the industry *did* eventually accept a tobacco tax increase to fund the Clinton health proposal, the tobacco documents also provide nine reasons why the industry believed the Clinton health proposal died [17, 37]. These reasons included election year politics [32, 34], Congressional attention to other issues [32, 34], lack of time for passage [34, 37], lack of cost estimates for alternative plans [21, 37], and conservative opposition [21, 27, 30, 34, 40]. Other key reasons that played a key role in the defeat of the Bill included opposition to employer mandates [15], higher future taxes [28, 29, 31, 33], and taxes on insurance plans [34]. Finally, the documents also illustrate that RJ Reynolds and Philip Morris, by far the two largest tobacco companies, did not think the 1994 legislation was necessarily dead and could well be revived in the 1995 session of Congress [17, 36]. As a November 1994 RJ Reynolds document explained:

‘Assumptions

- The Administration will bring forth another National Health Care Plan in 1995.
- “Clintoncare II” will begin being drafted between now and year-end, and will be completed early in the session. The President will speak to Health Care in his State of the Union Address as a goal for the ‘95 session.
- Changes in the makeup of the Senate and the House will make it more difficult to get massive, government-run Health Care Plan passed. Therefore, the plan will be less ambitious than “Clintoncare I,” perhaps in over several years. Some of the more controversial aspects, such as employer mandates, regional alliances, etc., could be eliminated, thereby appeasing some of the opponents that lined up against the Plan in 1994. If that’s the case, we lose many of the allies we had in ‘94.
- Universal coverage remains the goal’ [41].

An October 1994 Philip Morris document also noted in detail:

‘Early this summer (1994), the Senate Finance and the Senate Labor and Human Resources Committees favorably reported their versions of health care reform, and both Committees proposed a total tax increase of $1.00 per pack. Tobacco-state Democrats nonetheless were able to use their leverage as the congressional leadership searched for floor votes and incorporated the ‘45 cents over 5 years’ proposal into all final leadership bills. However, due to election year politics and intense opposition to comprehensive health care legislation, neither the House nor the Senate held floor votes on a health care package resulting in no tax increase in the 103^rd^ Congress. The Administration is already revamping its health care proposal for reintroduction in the 104^th^ Congress, and indications are that it will call for increased tobacco taxes to fund it’ [35].

Election year politics included, in particular, Republican opposition to national health reform as a key strategy of retaking Republican control of the Congress since the 1950s [44].

### Clinton White House documents

Previously secret and now declassified White House documents from 1993 to late 1994 present a clear picture of the extent and nature of the role of the tobacco tax in the defeat or not of the Health Security Act of 1994 [45–71]. In May 1993, First Lady Hillary Clinton and Miles Coggans, Special Assistant to the President on Agriculture, met with Senator Wendell H. Ford (D-Kentucky) to discuss the impact and a possible approach of mitigating the Administration’s proposed two dollar a pack increase in tobacco taxes to fund the Health Security Act of 1994 [50]. Before that meeting, an internal Administration memo noted:

‘An increase of $2.00 per pack of 20 in the federal excise tax would reduce U.S. cigarette consumption by 25 to 40 percent from 500 billion to 300 to 375 billion cigarettes. As a result, U.S. tobacco production could fall as much as 30 percent. To soften the impact of the decline in U.S. tobacco production, a proposal has been made to pay quota owners to retire unneeded quotas’ [50].

By December 1993, the Clinton Administration proposed that funding for the Health Security Act of 1994 come from a tobacco tax, Medicare and Medicare freezes and savings, corporate assessments, miscellaneous revenue gains, and savings in other federal programs [45–49, 51–55, 68].

In March 1994, following a meeting with Hillary Clinton and senior White House advisors, Carol Rasco, White House domestic policy advisor requested that Marion Berry, Agriculture Special Assistant to the President and Chris Jennings, White House health policy advisor work together on alternatives to the current funding proposal [57]. The purpose of this was to review Administration alternatives in light of members of Congress raising concerns about the amount of the proposed tobacco tax [57]. On the table for analysis was tobacco import quota or tariff relief such as the proposed Ford Amendment that limited foreign tobacco sales in the U.S. to 25% of the market and increased tariffs on imported foreign tobacco [57]. Another alternative that was analyzed was raising the tobacco excise tax from .24 cents to one dollar a pack instead of two dollars a pack. Related to this meeting and the current tobacco tax proposal an internal White House memo noted:

‘The tobacco industry obviously feels mistreated because of the single nature of the above taxes. Other taxes have been discussed with the President and he rejected them at the time. Some of these are taxes on various alcoholic beverages and soft drinks. They are still a possibility and would definitely soften the blow to the tobacco interests’ [57].

By June to July 1994, discussions by members of Congress and congressional committees regarding substantially reducing the proposed tobacco tax amount continued [58–60]. On 20 July 1994, Hillary Clinton met with 21 Democratic members of the U.S. House who were both supportive and ‘swing’ members to ensure their support of the Health Security Act [61]. Two issues that several in the group wished to have addressed were abortions and the tobacco tax [61]. Despite the concerns expressed at the meeting, the proposed tobacco tax tentatively remained at one dollar a pack [62, 63].

However and after the meeting, by July 25, 1994 the proposed tobacco tax had dropped from two dollars to one dollar to .45 cents a pack [62, 63]. Under the new proposal, funding was to come from a reduced tobacco tax, increased Medicaid and Medicare freezes and savings, corporate assessments, miscellaneous revenue gains, and savings in other federal programs [66].

After the compromise to reduce the tobacco tax to 45 cents a pack, in August 1994, a White House memo outlined numerous key issues other than tobacco that business interests had with the Health Security Act [13]. The memo noted:

‘I (Caren Wilcox) am continuing to work with the few elements of the large business community who remain committed to improving the bill sufficiently enough in the Senate that they believe a good bill could emerge from conference (committee). However, even these companies are expressing discouragement or concern. In addition, the small business coalition remains a constant support, but they have two issues of importance: the definition of the self employed, and the equal pricing issue for drugs sold to pharmacies’ [13].

In addition to individual business opposition from Pharmaceutical Research and Manufacturer’s Association and HIAA, another corporate coalition that opposed the bill in its entirety was: the U.S. Chamber of Commerce, NFIB, National Association of Manufacturers, Business Roundtable, Association of Private Pension and Welfare Plans, and the Employee Retirement Income Security Act Industry Committee [11, 13]. In particular the business coalition opposition focused on several issues with the bill including financing, multi-state issues, cost containment and managed care, cafeteria plans tax, independent contractors and self-employed issues, local pharmacy issues, and technical amendments to clarify the bill [13]. With respect to the financing issue:

‘Companies which offer insurance now, find it difficult to swallow a complete carve out for businesses with under 25 employees, on top of premiums already being voluntarily paid. They are concerned that this carve out will also lead to a cost shift in the system which will cause their premiums to go up’ [13].

Besides technical amendments, business opponents were concerned with, ‘Multi-state companies with uniform national standard company packages losing that standard, less than rigorous cost containment provisions to reverse increasing premium increases, and cafeteria benefit plans allowing benefits options and containing costs being replaced by pre-determined premiums’ [13]. Also, business opponents opposed the change in definition of an employee to include independent contractors who would be covered with increased health premium costs under the proposed law as well as small pharmacies concerned about equal treatment for wholesale pricing from large pharmaceutical manufacturers [13].

‘By October 1994, and after the defeat of the Health Security Act, a White House memo concluded:

The reforms we are considering can be categorized in three ways: (1) coverage protections for the currently insured, (2) access to insurance guarantees for all, and (3) market-based approaches to promote desirable competition among insurers. All three of these categories have been the focus of major pieces of legislation (Bentsen, Mitchell, Gephardt, Chafee, Dole, Rowland/Bilirakis, Cooper, and others) throughout the last two Congresses.

Although complex, the issues are not new. However, while most bills appear to have many of the same goals, the approaches and potential consequences vary widely.

The challenge of pursuing any insurance reform agenda is to ensure that it will be drafted and implemented correctly. If not, we risk not only unacceptably disrupting the current market and raising premiums to particularly influential constituencies, but undermining public confidence in our ability to move forward with future reforms. If we succeed in passing a strong set of reforms, we can make real and positive change to our currently quite flawed insurance market’ [13].

## Discussion

In summary, the White House clearly identified general business opposition to the Health Security Act along with running out of time and sub-optimal, weak, and conflicting bills as the primary reasons for the ultimate demise of the Health Security Act. Not included due to the earlier compromise of 45 cents a pack was ideologically diverse third party organizations allied with or utilized by the tobacco industry.

## Conclusion

The Clinton White House and tobacco industry documents indicate the primary reason for the health reform bill’s defeat was widespread business opposition to the legislation, but not the tobacco industry (or its third party organizational allies), which accepted a compromise 45 cents per pack increase phased in over five years to pay for the bill. Additionally, as noted in the tobacco industry document searches providing possible linkages between the tobacco industry and other businesses in defeating the bill (Table 1); the best available evidence is minimal that such linkages existed in a significant manner before and particularly after the 45 cents a pack compromise. This conclusion of business opposition as well as other factors leading to the bill’s defeat is in sync with a vast majority of historical analyses of the failed 1994 Clinton health bill. As shown in this paper, other factors included issues with: expanded employer mandates, bill running out of time, conflicting bills, employer mandates, higher taxes on insurance plans, impact on medical research and education, Congressional attention to other issues, election year politics, and possible future excise tax possibilities.

A primary lesson learned for anti-tobacco advocates related to the lack of a role of the tobacco industry in the ultimate defeat of the health bill is that advocacy strategies and tactics can change at any point in the policy process. The tobacco industry’s early mobilization in tandem with third party allies ended prior to the defeat of the bill due to the 45 cents a pack political compromise. Anti-tobacco advocates need to carefully and continually document and assess such corporate or any other alliances with the industry throughout a policy process in anti-tobacco campaigns, but never assume such alliances exist at any point without hard evidence. Moreover, the tobacco industry was pressured to agree to a modest 45 cents a pack tax compromise in lieu of earlier legislative proposals of much higher tobacco pack tax increases. Thus, another primary lesson learned is that the industry can be pressured to settle for a compromise by starting from a position of negotiating strength when it sees the compromise in its interest and even contrary to more robust corporate profits. While the impact of tobacco use in the U.S. remains a significant preventable public health issue with over 480,000 annual deaths due to smoking and secondhand smoke this can be countered by higher tobacco taxes [72]. The compromise of 45 cents a pack would have been a modest victory for public health. Thus, anti-tobacco advocates should always be cognizant of their ultimate goal of reducing tobacco use when advocating or negotiating for compromises with the industry or others such as occurred with the Clinton health bill.

This study has the following limitations. Other evidence or documents could be available beyond this study documenting further insights into the policy advocacy of third party organizations in alliance with or utilized by the tobacco industry from 1993 to 1994. This could provide further information on the dynamics of the overall policy making processes leading to the defeat of the Clinton health bill. Similarly, there could be documents outside of the 1993–1994 time frame of this research that examined the legislative session in which the Clinton health care bill was defeated. This could provide further information and background on the dynamics of the tobacco industry in league with third party organizations and their role with respect to the Clinton health bill. Finally, while the methodology used in this study crosschecked the data and conclusions using two separate data sources including a recommended search term approach by an archivist at the Clinton Presidential Library. It is possible with respect to on-line document searches and due to the large number and types of documents in both archives there may be further relevant documents.

## Abbreviations

CDC: Centers for Disease Control and Prevention
HIAA: Health Insurance Association of America
HSA: Health Security Act
NFIB: National Federation of Independent Business

## Footnotes

1 In the article: 1. Tesler L, Malone R. “Our Reach is Wide by Any Corproate Standard”: How the Tobacco Industry Helped Defeat the Clinton Health Plan and Why It Matters Now. American Journal of Public Health. 2010;100 (7):1174–88., there is a detailed overview of this manufactured grassroots lobbying effort. This eventually led to the 45 cents increase over five years compromise agreed to by the tobacco industry in August 1994.
